# A machine learning COVID-19 mass screening based on symptoms and a simple olfactory test

**DOI:** 10.1038/s41598-022-19817-x

**Published:** 2022-09-16

**Authors:** Youcef Azeli, Alberto Fernández, Federico Capriles, Wojciech Rojewski, Vanesa Lopez-Madrid, David Sabaté-Lissner, Rosa Maria Serrano, Cristina Rey-Reñones, Marta Civit, Josefina Casellas, Abdelghani El Ouahabi-El Ouahabi, Maria Foglia-Fernández, Salvador Sarrá, Eduard Llobet

**Affiliations:** 1 Servei d’Urgències, Hospital Universitari Sant Joan, Reus, Spain; 2 Sistema d’Emergències Mèdiques de Catalunya, Barcelona, Spain; 3 Institut d’Investigació Sanitària Pere i Virgili (IISPV), Tarragona, Spain; 4 Departament d’Enginyeria Química, Universitat Rovira i Virgili, Tarragona, Spain; 5 CUAP Reus, Gerència Territorial Camp de Tarragona, Institut Català de la Salut, Tarragona, Spain; 6 Research Support Unit-Camp de Tarragona, Catalan Institute of Health (ICS), Tarragona, Spain; 7 School of Medicine and Health Sciences, Universitat Rovira i Virgili, Reus, Spain; 8 Atenció Primaria CAP Maria Fortuny-Reus V, Reus, Spain; 9 ORL Service Sant Joan University Hospital, Reus, Spain; 10 Direcció Médica, Hospital Universitari Sant Joan, Reus, Spain; 11 MINOS-IURESCAT, Universitat Rovira i Virgili, Tarragona, Spain; 12 IDIAP Jordi Gol, Catalan Institute of Health (ICS), USR Camp de Tarragona, Reus, Spain

**Keywords:** Population screening, Viral infection

## Abstract

The early detection of symptoms and rapid testing are the basis of an efficient screening strategy to control COVID-19 transmission. The olfactory dysfunction is one of the most prevalent symptom and in many cases is the first symptom. This study aims to develop a machine learning COVID-19 predictive tool based on symptoms and a simple olfactory test, which consists of identifying the smell of an aromatized hydroalcoholic gel. A multi-centre population-based prospective study was carried out in the city of Reus (Catalonia, Spain). The study included consecutive patients undergoing a reverse transcriptase polymerase chain reaction test for presenting symptoms suggestive of COVID-19 or for being close contacts of a confirmed COVID-19 case. A total of 519 patients were included, 386 (74.4%) had at least one symptom and 133 (25.6%) were asymptomatic. A classification tree model including sex, age, relevant symptoms and the olfactory test results obtained a sensitivity of 0.97 (95% CI 0.91–0.99), a specificity of 0.39 (95% CI 0.34–0.44) and an AUC of 0.87 (95% CI 0.83–0.92). This shows that this machine learning predictive model is a promising mass screening for COVID-19.

## Introduction

Since the first cases of severe acute respiratory syndrome coronavirus 2 (SARS-COV-2) were diagnosed in December 2019 in the Chinese city of Wuhan, the coronavirus disease 2019 (COVID-19) has spread rapidly^1^. The strategies applied in the vast majority of countries to control the virus transmission have been ineffective. The first results concerning the safety and effectiveness of different types of vaccines have raised optimism in the scientific community due to the possibility of controlling COVID-19^2^. But recent real-world data indicate that the effectiveness of the BNT162b2 and ChAdOx1 vaccines against infections with symptoms or high viral burden is reduced with new variants such as delta. In addition, infections in vaccinated patients have similar viral loads compared to unvaccinated patients^3^. This justifies the high number of new cases and a mortality rate difficult to eradicate in some countries with high vaccination rates^4^. On the other hand, a large part of the world population remains susceptible to SARS-COV2 infection, due to lack of access to vaccines, making herd immunity likely unachievable.

The early detection of symptoms suggestive of infection, rapid and efficient testing, contact tracing and isolation are the basis of an effective screening strategy to control transmission of COVID-19 and decrease the disease burden on healthcare systems. The Achilles' heel in the fight against this disease is the large number of patients who are asymptomatic or have only a few symptoms that are difficult to differentiate from a common cold, but who are nonetheless able to transmit the disease^5,6^. It was estimated that 51.9% of SARS-COV-2 infected cases were asymptomatic or had only 1 or 2 symptoms suggestive of COVID-19^7^. The reference diagnostic tool for COVID-19 is reverse transcriptase polymerase chain reaction (RT-PCR). Its accessibility may be limited for low-resource healthcare systems and its cost and time requirements preclude its use as a mass triage tool. Recently a screening tool based on a machine learning model including clinical features and symptoms has been constructed to prioritize testing for COVID-19^8^. It was found that a predictive model for COVID-19 that included the combination of symptoms and wearable sensor data performed better than a model based on symptoms alone^9^.

Olfactory dysfunction (OD) has recently been described as one of the most prevalent symptoms reaching 50% to 75% of COVID-19 patients and could be used as a means of screening to help identify people who should self-isolate^10–12^. With the delta variant OD has been described in the 39% of the cases being as well one of the most prevalent symptom^13^.

A symptom predictive model for COVID-19 based on a smartphone app including age, sex, loss of smell and taste, persistent cough, severe fatigue and skipped meals obtained a sensitivity of 65%^14^. At the time of diagnosis, a recent prospective study found that 31% of patients affected by COVID-19 presented OD^15^. Between 11.8 and 23% of cases presented OD before any other symptoms^10,16^. The validated olfactory tests are subjective and difficult to implement. A recent study showed that a simplified test based on the identification and the assessment of intensity of three different scents was able to detect unperceived OD in COVID-19 patients^15^.

Hydroalcoholic gels are widely distributed as they are one of the main strategies for decreasing virus transmission^17^. Fragrance essential oils such as lavender, eucalyptus and lemon make them more pleasant and can enhance their anti-viral effect^18,19^. These features make an aromatized hydroalcoholic gel a good candidate for being used as part of a simple, fast and cost-effective large-scale olfactory screening test.

The aim of this study was to develop and validate, using cross-validation techniques, a machine learning diagnostic predictive model for COVID-19 mass screening using symptoms and a simple olfactory test based on an aromatized hydroalcoholic gel, which could be especially useful when testing resources are limited.

## Results

### Characteristics of the study population

**Figure 1:**
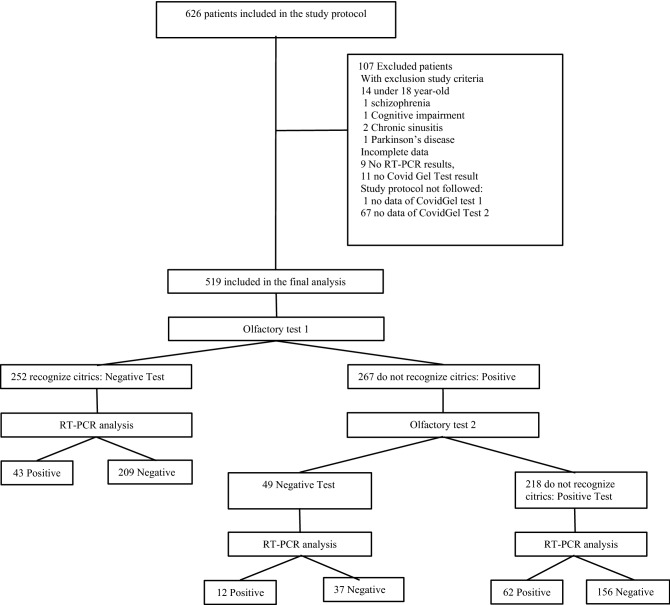
Flow chart.

During the study period 3788 patients underwent RT-PCR to diagnose COVID-19 at one of the study health centres. The inclusion of cases and RT-PCRs performed per week at the centres while participating in the study can be consulted in the supporting information Fig. S1. A total of 626 patients were initially included in the study protocol. Of these, 107 patients were excluded because of incomplete data or exclusion criteria as shown in Fig. 1.

The final analysis of the study included 519 patients, out of whom 341 patients (65.7%) were from primary care and 179 (34.3%) were from the hospital Emergency Department. According to the criteria for carrying out a RT-PCR test, 386 (74.4%) had at least one symptom suggestive of COVID-19, 118 (22.7%) were asymptomatic and were close contacts of a COVID-19 case, and 15 (2.9%) were asymptomatic and were tested for unknown reasons. A positive RT-PCR was found in 117 patients (22.5%) and a negative RT-PCR was found in 402 patients (77.5%).

The mean (SD) age of the study population was 42.3 (16.3) years, the age range was between 18 and 98 years and 48% were male. None of the patients requiring hospital admission died. Table 1 shows the background and clinical characteristics of the study population.

**Table 1:** Background and clinical characteristics of the study population.

Variable	Total	SARS-CoV2 positive	SARS-CoV2 negative	
	N = 519	N = 117	N = 402	Absolute difference (95% CI). %
**Demographic data**				
Male patients	249 (48)	68 (58.1)	181 (45)	13.09 (2.92 to 23.27)
Age (years)	42.3 (16.3)	43.4 (15.95)	41.9 (16.3)	1.51 (− 1.82 to 4.83)
**Background**				
Hypertension	96 (18.8)	24 (20.9)	72 (18.2)	2.64 (− 5.7 to 10.99)
Diabetes	39 (7.6)	9 (7.8)	30 (7.6)	0.23 (− 5.33 to 5.79)
Dyslipidaemia	57 (11.2)	16 (13.9)	41 (10.4)	3.53 (− 3.47 to 10.54)
Smoking	82 (16.1)	11 (9.6)	71 (18)	− 8.41 (− 14.98 to − 1.83)
Enolism	18 (3.5)	8 (7)	10 (2.5)	4.42 (− 0.48 to 9.33)
Chronic bronchopathy	60 (11.8)	13 (11.3)	47 (11.9)	− 0.59 (− 7.2 to 6.02)
Chronic heart disease	28 (5.5)	5 (4.3)	23 (5.8)	− 1.47 (− 5.86 to 2.91)
Neoplasia	19 (3.7)	4 (3.5)	15 (3.8)	− 0.32 (− 4.16 to 3.52)
Autoimmune disease	14 (2.7)	6 (5.2)	8 (2)	3.19 (− 1.1 to 7.49)
Chronic renal failure	4 (0.8)	0 (0)	4 (1)	− 1.01 (− 2 to − 0.03)
Chronic liver disease	14 (2.7)	5 (4.3)	9 (2.3)	2.07 (− 1.94 to 6.08)
Hypothyroidism	26 (5.1)	3 (2.6)	23 (5.8)	− 3.21 (− 6.93 to 0.5)
Obesity	43 (8.3)	11 (9.4)	32 (8)	1.44 (− 4.47 to 7.35)
Chronic cortico-therapy	16 (3.1)	11 (9.6)	5 (1.3)	8.3 (2.81 to 13.79)
Immunosuppressive therapy	5 (0.98)	2 (1.7)	3 (0.8)	0.96 (− 1.55 to 3.48)
**Disease severity**				
Mild	334 (64.5)	74 (63.2)	260 (64.8)	− 1.59 (− 11.5 to 8.32)
Moderate	30 (5.8)	22 (18.8)	8 (2)	16.81 (9.6 to 24.02)
Severe	6 (1.2)	2 (1.7)	4 (1)	0.71 (− 1.83 to 3.25)
Oxygen therapy	38 (7.34)	21 (17.9)	17 (4.2)	13.71 (6.48 to 20.94)
**Diagnosis**				
Upper respiratory tract infection	66 (12.7)	34 (29.1)	32 (8.0)	21.1 (12.5 to 29.7)
Lower respiratory tract infection	22 (4.2)	2 (1.7)	20 (5.0)	− 3.3 (− 6.4 to − 0.10)
Pneumonia	28 (5.4)	21 (18.0)	7 (1.7)	16.2 (31.5 to 50.5)

Values are median (Standard Deviation) and n (%).

### COVID-19 symptoms and olfactory test results

The mean (SD) number of days of the symptom evolution was 5.8 (5.6) for the COVID-19 positive patients and 5.1 (12.1) for the COVID-19 negative patients with an absolute difference of 0.75 (95% CI − 1.35 to 2.84; P = 0.48). The symptoms most strongly associated with COVID-19 were OD and GD. Fever, dry cough, asthenia, myalgia, headache, diarrhoea, OD, and GD were the eight symptoms associated with COVID-19. Table 2 shows the reported symptoms and olfactory test results in the population study.

**Table 2:** Patient reported COVID-19 symptoms and olfactory test results.

	SARS-COV2 positive	SARS-COV2 negative		P-value
	N = 117	N = 402	Odds ratio (95% CI)	
**Symptoms**				
Fever	59 (50.4)	101 (25.1)	3.03 (1.98–4.65)	0.00
Dry cough	45 (38.5)	73 (18.2)	2.82 (1.8–4.42)	0.00
Asthenia	34 (29.1)	60 (14.9)	2.33 (1.44–3.79)	0.00
Myalgias	30 (25.6)	61 (15.2)	1.93 (1.17–3.17)	0.01
Cephalea	39 (33.3)	83 (20.6)	1.92 (1.22–3.03)	0.01
Diarrhoea	35 (29.9)	82 (20.4)	1.67 (1.05–2.65)	0.04
OD	19 (16.2)	13 (3.2)	5.79 (2.76–12.12)	0.00
GD	25 (21.4)	18 (4.5)	5.78 (3.03–11.04)	0.00
Dyspnoea	23 (19.7)	54 (13.4)	1.58 (0.92–2.7)	0.13
Productive cough	15 (12.8)	40 (10)	1.33 (0.7–2.5)	0.48
Sore throat	31 (26.5)	96 (23.9)	1.15 (0.72–1.84)	0.65
Rhinorrhoea	6 (5.1)	9 (2.2)	2.36 (0.82–6.77)	0.18
Anorexia	10 (8.5)	30 (7.5)	1.16 (0.55–2.45)	0.85
Asymptomatic	13 (11.1)	120 (29.9)	0.29 (0.16–0.54)	0.00
**Symptoms combination**				
GD and OD	31 (26.5)	24 (6)	5.66 (3.16–10.13)	0.00
Fever and dry cough	75 (64.1)	146 (36.3)	3.13 (2.04–4.81)	0.00
Fever, dry cough and OD	82 (70.1)	152 (37.9)	3.84 (2.46–5.98)	0.00
**Olfactory test results**				
Test 1 positive	74 (63.2)	193 (48)	1.86 (1.22–2.85)	0.01
No smell at all	13 (11.1)	12 (3)	4.06 (1.8–9.17)	0.00
Test 1 and 2 positive	62 (52.9)	156 (38.8)	1.78 (1.17–2.69)	0.01

*OD* Olfactory dysfunction, *GD* Gustatory dysfunction, Values are n (%).

In the total population study, the olfactory test 1 was positive in 267 patients (51.4%) and negative in 252 patients (48.6%). Among patients with a positive olfactory test result 112 cases (41.9%) identified the gel smell as alcohol, 57 cases (21.3%) as cologne, 27 cases (10.1%) as aromatic herbs, 10 cases (3.7%) as non-citrus fruits (3.7%), 6 cases (2.2%) as alcoholic beverages (2.2%) and 22 cases (8.2%) as other responses. In 25 cases (9.4%) participants reported that they “didn’t smell anything at all” and in 8 cases a “don’t know” response (3%) was reported. Among patients with a negative olfactory test result, 207 cases (82.1%) identified the gel smell as lemon, 26 cases (10.3%) as citrus, 13 cases (5.1%) as orange, 2 cases (0.8%) as tangerine, 2 cases (0.8%) as citronella, and 2 cases (0.8%) as lime.

A positive olfactory test 1 was associated with COVID-19 (OR 1.86; 95% CI 1.22–2.85, P < 0.01). The response “do not smell anything at all” was strongly associated with COVID-19 (OR 4.06; 95% CI 1.8–9.17). Among the 13 asymptomatic COVID-19 positive patients, 10 (76.9%) had a positive olfactory test 1 result and only 3 patients presented a negative olfactory test 1. An olfactory test 1 positive result in asymptomatic patients was associated with COVID-19 (OR 3.94; 95% CI 1.03–15.03). The detailed results of the olfactory test and the diagnostic values of the relevant symptoms, the combination of symptoms and olfactory test for predicting COVID-19 were available in the S1 and S2 Tables.

### Results of the machine learning predictive model

Table 3 shows the results of the different classification trees constructed with machine learning according to the variables introduced in the model.

**Table 3:** Results of machine learning model.

	Sensitivity (95% CI)	Specificity (95% CI)	PPV (95%CI)	NPV (95%CI)	BA	F1	MCC	AUC (95% CI)
**Sensitive tree**								
Relevant symptoms	*0.86 (0.79–0.92)*	*0.37 (0.33–0.42)*	*0.29 (0.24–0.34)*	*0.9 (0.85–0.94)*	*0.62*	*0.43*	*0.21*	*0.86 (0.81–0.9)*
	**0.97 (0.92–0.99)**	**0.11 (0.07–0.15)**	**0.29 (0.24–0.34)**	**0.91 (0.76–0.98)**	**0.54**	**0.44**	**0.12**	**0.89 (0.84–0.93)**
Relevant symptoms and olfactory test	*0.94 (0.88–0.98)*	*0.32 (0.28–0.37)*	*0.29 (0.24–0.34)*	*0.95 (0.9–0.98)*	*0.63*	*0.44*	*0.25*	*0.87 (0.83–0.92)*
	**0.96 (0.9–0.99)**	**0.23 (0.18–0.28)**	**0.32 (0.26–0.37)**	**0.94 (0.86–0.98)**	**0.60**	**0.48**	**0.22**	**0.89 (0.84–0.93)**
Relevant symptoms. olfactory test. sex and age	*0.97 (0.91–0.99)*	*0.39 (0.34–0.44)*	*0.31 (0.27–0.36)*	*0.97 (0.94–0.99)*	*0.68*	*0.47*	*0.32*	*0.87 (0.83–0.92)*
	**0.98 (0.93–1)**	**0.31 (0.26–0.37)**	**0.34 (0.29–0.4)**	**0.98 (0.92–1)**	**0.64**	**0.51**	**0.30**	**0.89 (0.84–0.93)**
**Specific tree**								
Relevant symptoms and age	*0.29 (0.21–0.38)*	*0.95 (0.92–0.97)*	*0.62 (0.48–0.75)*	*0.82 (0.78–0.85)*	*0.62*	*0.40*	*0.32*	*0.85 (0.8–0.89)*
	**0.33 (0.24–0.43)**	**0.93 (0.89–0.95)**	**0.62 (0.48–0.75)**	**0.79 (0.74–0.83)**	**0.63**	**0.43**	**0.32**	**0.82 (0.77–0.87)**

*AUC* Area under the curve, *BA* Balanced Accuracy, *MCC* Matthews correlation coefficient.

The italic rows show the total population study and the bold rows show symptomatic patients.

By only introducing the relevant symptoms into the model, the sensitivity was 0.86 (95 CI 0.79–0.92), the specificity was 0.37 (95% CI 0.33–0.42) and the AUC was 0.86 (0.81–0.9) for the total population study, and 0.97 (95%CI 0.92–0.99), 0.11 (95% CI 0.07–0.15) and 0.89 (95% CI 0.81–0.9) respectively for the symptomatic population. The sensitivity and specificity obtained was 0.94 (95%CI 0.88–0.98) and 0.32 (95% CI 0.28–0.37) when the olfactory test was introduced into the model for the total study population. The constructed sensitive classification tree only took into account the result of the olfactory test 1 and ignored the result of the olfactory test 2. Considering other clinical variables, the model also included sex and age, reaching a sensitivity of 0.97 (0.91–0.99), a specificity of 0.39 (0.34–0.44) and an AUC of 0.87 (95% CI 0.83–0.92) for the total study population, and 0.98 (95%CI 0.93–1), 0.31 (95% CI 0.26–0.37) and 0.89 (95% CI 0.84–0.93) for the symptomatic population, respectively. The specific classification tree built took into account the relevant symptoms and age and obtained a sensitivity of 0.29 (0.21–0.38), a specificity of 0.95 (95% CI 0.92–0.97) and an AUC of 0.85 (0.8–0.89) for the total study population.

**Figure 2:**
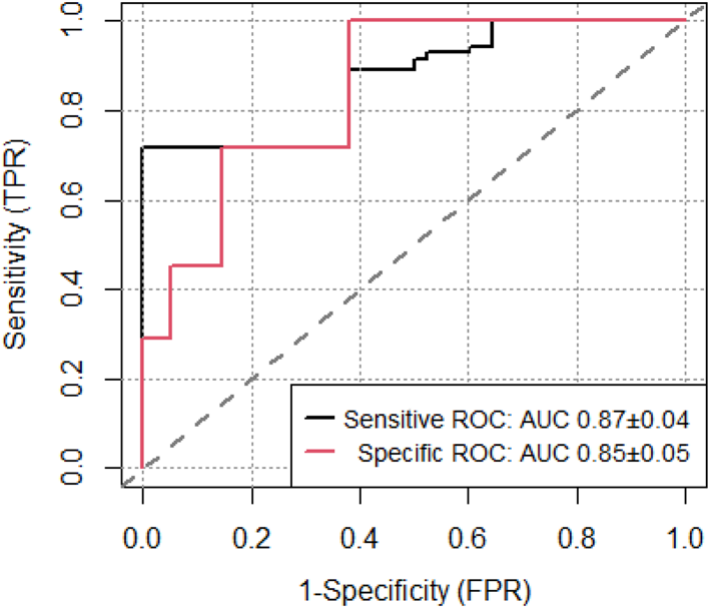
Sensitive and specific classification tree algorithm ROC curve.

The resulting receiver operating characteristic (ROC) curve is shown in the Fig. 2 and the precision-recall curve of the sensitive and specific tree algorithm are shown in Figs. S2 and S3.

## Discussion

The combination of symptoms and a simple olfactory test based on identifying the smell of a hydroalcoholic gel made it possible to develop a predictive model with high sensitivity, which has important clinical implications.

A predictive model based on symptoms reported on a smartphone-based app obtained a lower sensitivity of 0.65 (95% CI 0.62–0.67) and a lower AUC of 0.76 (95% CI 0.74–0.78) to predict COVID-19 than our predictive model^14^. Another predictive model using machine learning based on symptoms, gender, age and close contacts obtained a lower sensitivity which was between 0.85 and 0.87 depending on the possible working points and a similar AUC of 0.86 (95% CI 0.85–0.87)^8^. The different results of our model, depending on the variables included, show similar or even higher diagnostic values with respect to those models proposed as population screening. The model presented has the advantage that it includes asymptomatic patients and does not include close contacts in its variables as this could be difficult to determine in a situation of community transmission. To our knowledge, this is the first model including an olfactory test and built using a prospective population-based study**.** It is important to highlight that the symptoms combination have the higher weight in the model results, although the low false negative rate of the olfactory test among asymptomatic COVID-19 patients, helps improving the sensitivity of the model. The sample for this population-based study was obtained from patients following current indications for RT-PCR testing, and the sensitivity and specificity figures obtained make this model useful as a population-based screening.

In the same direction, new advances are being made in the development of new point-of-care, rapid, sensitive and inexpensive diagnostic methods to detect COVID-19 that can be useful to fight this pandemic and prepare for the next ones^20^. An effective mass screening based on antigen test detection of SARS-CoV-2 has been described^21^. But a recent review of several antigen tests on the market states that two-thirds had overall sensitivities (30.8%-68.9%) below the World Health Organization recommended standard of ≥ 80% raising concerns whether the antigen detection alone is sufficient for COVID-19 mass screening^22^. Combining our predictive model with an antigen test could be a promising mass screening strategy.

Regarding the olfactory test, it has obtained a sensitivity almost twice as high as a more complex olfactory test for predicting COVID-19 based on identifying the smell of three scented paper strips and a 4-item scale intensity rate^15^. In addition, the simplicity of the olfactory test means it can be implemented as a self-test, making it a more suitable population screening olfactory test than any test reported so far. The wide distribution of this predictive tool due to its low cost also contributes to improving the disease situational awareness of the population. This may be especially useful in those scenarios where preventive measures are gradually being relaxed and there is still a need to protect older and more vulnerable people due to the rapid waning of vaccine protection over time against new variants such as omicron^23,24^.

Our work has some limitations. Our study was conducted when the alpha variant was the most predominant variant in our area. Recent data show a high prevalence of classical symptoms such as cough, fever and olfactory and taste dysfunction among vaccinated and unvaccinated COVID-19 infected patients where the delta variant is predominant suggesting that our model may be useful in this setting^3^. The omicron variant has been associated with a reduced capacity to penetrate olfactory epithelial cells and produce anosmia^25^. A prospective study based on a focused questionnaire for assessing olfactory function found that the prevalence of OD caused by the omicron variant was 24.6%^26^. This drop in the prevalence of OD with this variant may affect the sensitivity of our model. Regarding the olfactory test, a high percentage of patients identified the smell of the gel as alcohol. The alcoholic matrix of the gel could hinder olfactory recognition, explaining the low specificity found. Moreover, the patient’s capacity to identify smell may decrease in an uncomfortable scenario such as an emergency department during a pandemic.

It is important to highlight that in our study no side effects related to the inhalation of the hydroalcoholic gel were reported. One study described that repeated exposure to a hydroalcoholic gel by inhalation does not increase blood ethanol levels^27^. The side effects described in the literature are related to the occurrence of dermatitis or are due to the ingestion of the gel^28,29^.

The value added by our COVID-19 predictive model in this field is its potential applications such as its inclusion in a mass testing strategy in order to save costs. Our predictive model could be useful to quickly rule out non-infected patients and for selecting the population that could benefit from a more expensive diagnostic test such as antigen testing or RT-PCR helping to reduce the costs for the health system or for companies with a rigorous occupational risk policy such as hospitals, nursing homes or large companies. It could also be especially useful for controlling transmission in those regions where testing resources are limited due to scarce economic resources or logistical difficulties.

This predictive model has been patented (EP 21 382 524.3) and is available upon request. The effectiveness of its implementation in different epidemiological settings should be tested by performing external validations; therefore, the collaboration of the scientific community is encouraged.

## Conclusion

A machine learning predictive model for COVID-19 using symptoms and a simple olfactory test based on an aromatized hydroalcoholic gel showed high sensitivity for diagnosing COVID-19. The capacity of this predictive model to detect infected SARS-COV-2 patients among asymptomatic patients makes it a promising tool for the fight against COVID-19. This predictive model could be especially useful for mass screening when testing resources are limited.

## Methods

### Study design and setting

This is a population-based prospective cohort study conducted following the TRIPOD Statement for multivariable diagnosis prediction model^30^. The study was carried out in the Emergency Department of Sant Joan University Hospital of Reus, which is the reference hospital of the region, and in all five primary care centres of Reus (Catalonia, Spain) public health network.

The municipality of Reus is located in the Mediterranean area, has a surface area of 52.82 km^2^ and at the beginning of 2020 it had a population of 106,168 inhabitants and a density of 2010.0 inhabitants/km^2^^31^. This study was approved by the Ethics Committee of the Pere i Virgili Health Research Institute (Ref: 120/2020) and the IDIAP Jordi Gol Clinical Research Ethics Committee (Codi: 20/114-PCV). The study was conducted in accordance with de Declaration of Helsinki and Good Clinical Practices. All study participants were required to sign an informed consent form.

### Participants

The study included consecutive patients undergoing RT-PCR for the first time to rule out COVID-19 infection who consulted the hospital emergency department or their primary care centre between 15 June and 11 September, 2020. Patients were tested for presenting symptoms suggestive of COVID-19 or for being close contacts of a confirmed COVID-19 case. Close contacts were considered those persons who had shared an area with a positive case at a distance of less than 2 m, for more than 15 min, without protection and from 48 h prior to the onset of symptoms.

The study did not include patients under 18 years of age, patients who did not sign the informed consent form, and patients with pathologies or conditions that may interfere with the olfactory function, such as any degree of cognitive impairment, Parkinson's disease, chronic rhinosinusopathy, head trauma, nasal obstruction, treatment with high concentrations of oxygen, acute respiratory failure, patients with an altered state of consciousness, or who use inhaled corticosteroids.

### Olfactory test development

A multidisciplinary cooperation was established for creating a hydro-alcoholic hand sanitizing gel that meets current requirements in terms of its composition^32^.

Based on the literature and habits of our Mediterranean study population, it was determined that the most suitable odoriferous substance was lemon^33^. Tests were carried out with different concentrations of lemon essential oil and lemon fragrances of synthetic origin. The composition of the gel was adapted to attenuate the smell of alcohol. A study was carried out to determine the most effective composition with and without thickener. Gas chromatography and mass spectrometry were used to obtain semi-quantitative results. A headspace sampling technique was used to establish the effectiveness of the volatile odoriferous substance that evaporated from the hydroalcoholic gel at 37 ºC. Finally, two hydroalcoholic gels with increasing concentrations of lemon essential oil were created as an olfactory test.

### Description of the olfactory test

The olfactory test was performed by appropriately trained primary care and emergency nurses before the sample for SARS-COV-2 RT-PCR was collected. Therefore, both the patient and the healthcare personnel did not know the patient’s infection status. Firstly, the test consisted of applying 1 ml of 0.3% gel (olfactory test 1) using a dispenser onto the patient’s palm. Then the patient rubbed the gel on their hands and waited for 3 s. The patient was then asked to smell their hands and to “please, identify the smell of this gel”. The answer was recorded on the basic data collection sheet regardless of the result. If the answer was not lemon or if it was inconclusive, the same test was repeated after 30 s with the 0.5% gel (olfactory test 2). The olfactory test was considered negative if the patient recognized a citrus fruit, and the olfactory test was considered positive if the patient could not smell the gel or did not recognize a citrus fruit.

### Data collection

A data collection sheet was completed by the attending nurse before taking the sample for the RT-PCR test. It included the results of the two olfactory tests when both were performed. It also included age, gender, duration of symptoms (in days), and a yes/no questionnaire to check for symptoms such as fever, dry cough, dyspnoea, anorexia, myalgia, headache, diarrhoea, asthenia, productive cough, sore throat, OD or gustatory dysfunction (GD), others or no symptoms. The RT-PCR test for detecting SARS-COV-2 was considered the gold standard for diagnosis. During our study, the RT-PCR was performed by trained personnel according to the technical considerations of the manufacturer using a double sampling of the pharynx and the nose. The conservation of the sample and the transfer to the laboratory followed the channels of the usual clinical practice of the centre. RT-PCR tests were carried out with the VIASURE SARS-COV-2 Real Time PCR Detection Kit (CerTest Biotec, Zaragoza, Spain), or with the Procleix1 method in a Panther automated extractor and amplifier (Grifols Laboratories, Barcelona, Spain). Once all the data collection sheets were completed, the medical digital records were consulted and the RT-PCR test results were recorded, as well as the patient’s background, evolution and discharge diagnosis. Regarding the severity of the disease, the patients attended and discharged immediately were considered as mild, those admitted to the hospital as moderate and those requiring ICU during hospitalization as severe.

This study was conducted at the beginning of the second wave of COVID-19 in our region^34^. The 14-day cumulative incidence of COVID-19 cases in the city of Reus increased gradually from 0.9 cases/100,000 inhabitants on 15 June to 376.09 cases/100,000 inhabitants on 24 August^35^.

### Model development and internal validation

First, an analysis was conducted to explore the independent variables associated with COVID-19. The symptoms that proved to be statistically significant in a logistic regression predictive model, were fever, dry cough, myalgia, headache, diarrhoea, asthenia, altered sense of smell, and altered sense of taste. These 8 symptoms were defined as relevant and presented as well as their combinations the strongest associations with the predicted event. Diagnostic values were calculated for each symptom separately and their combinations for the total population and the symptomatic population. The productive cough variable was also included as a relevant symptom.

In order to facilitate the search for the best combination of variables to predict the diagnosis of COVID-19, we decided to build a model based on a decision tree constructed by machine learning that could also facilitate its clinical use following guidelines^36^. Other modelling methodologies such as random forests or artificial neural networks were discarded because they need larger training datasets and also because their interpretability is not as straightforward as that of decision trees. Priority was given to the construction of a parsimonious model using as few variables as possible, robust by minimising missing data, transparent and simple. Moreover, minimising false negatives was also a priority in the predictive model construction to allow its use as a population screening.

The 8 relevant symptoms and the result of the olfactory test were variables significantly associated with COVID-19. Sex and age were as well sequentially introduced into the model as these variables were considered clinically relevant^10^. The final model had 11 independent variables therefore the study sample complied with the standard rule of ten clinical events per predictive variable^37^.

The number of relevant symptoms was counted for each patient and this new variable was used to develop the model based on classification trees using a recursive partitioning algorithm^38^. The growth of the trees was controlled to avoid overfitting the data. Trees were pruned to the size that minimized the cross-validated error. In addition, these classification trees were built using the following parameters: the splitting index was the Gini coefficient; the minimum number of patients in any node of a tree for a split to be attempted was set at 30; the minimum number of patients in any terminal node of a tree was set at 10; node splits were only attempted if they improved the fit by a factor of 0.01; and the number of cross-validations to be run was set at 10. The sizes of the trees obtained using this strategy range between six and seven leaves (terminal nodes), which proves that overfitting has been successfully avoided.

In order to obtain different values of sensitivity and specificity in the resulting classification trees, distinct costs of false positives and false negatives were used in the loss matrix parameter that drives the splitting function of the classification tree algorithm. In particular, the specific classification tree was grown using equal cost values for false positives and false negatives, while the sensitive classification tree was grown using a cost value for false negatives that was eight times the cost value for false positives.

The internal model validation was carried out using the R package cross validation techniques in machine learning.

### Statistical analysis

The quantitative variables used in this study were described using the mean, the standard deviation (SD), the median and the first and third quartiles. The differences between means and their corresponding 95% confidence interval (CI) were also used to compare groups of patients. Categorical variables were described using the number of cases, percentages and 95% CI. Comparisons between groups of patients were performed using Student’s T test for quantitative variables, while the chi-squared test was used for categorical variables. Groups of patients were also compared in terms of the risk difference and odds ratio (OR) of the binary variables, and their corresponding 95% CI. All tests were two-tailed and P-values lower than 0.05 were considered statistically significant. Diagnostic values in terms of sensitivity, specificity, positive predictive value, negative predictive value, positive likelihood ratio and negative likelihood ratio, as well as their corresponding 95% CI, were calculated for the binary variables and smell tests. Several predictive models were analysed to handle missing data in the study protocol. A data-complete analysis was adopted over other strategies due to the low relevance of the missing data in the final results of the predictive machine learning model. All statistical analyses were performed using R software version 4.0.

## Supplementary Information

Supplementary Information.

## Data Availability

The datasets generated and/or analysed during the current study are not publicly available but are available from the corresponding author on reasonable request.
