# Supplementary material for: A machine learning COVID-19 mass screening based on symptoms and a simple olfactory test
Source: Sci Rep. 2022 Sep 16;12:15622. doi: 10.1038/s41598-022-19817-x (PMC9481525; doi:10.1038/s41598-022-19817-x)
Supplement: Supplementary file 1 — Supplementary Information. [file 41598_2022_19817_MOESM1_ESM.docx]

**Supplementary Information**

**S1 Fig.** Case inclusion and RT-PCRs performed per week and 14-day cumulative incidence of COVID-19 cases in the city of Reus during the study period.

**S2 Fig.** Precision-recall curve for the sensitive tree algorithm.

**S3 Fig.** Precision-recall curve for the specific tree algorithm.

**S1 Table.** Olfactory test in relation to symptoms.

**S2 Table.** Relevant symptoms and Olfactory Test diagnostic values

**S1 Fig.** **The case inclusion and RT-PCRs performed per week and 14-day cumulative incidence of COVID-19 cases in the city of Reus during the study period.**


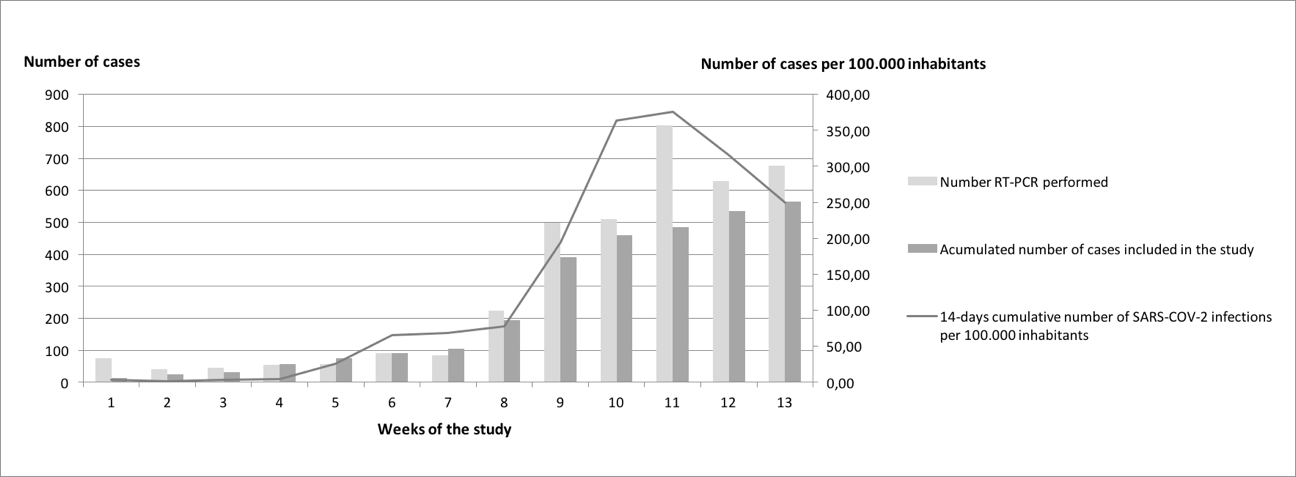


**S2 Fig. Precision-recall curve for the sensitive tree algorithm.**


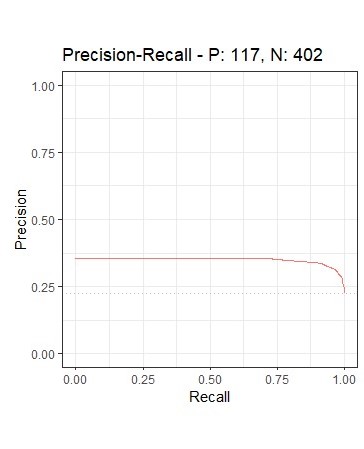


**S3 Fig.** **Precision-recall curve for the specific tree algorithm.**


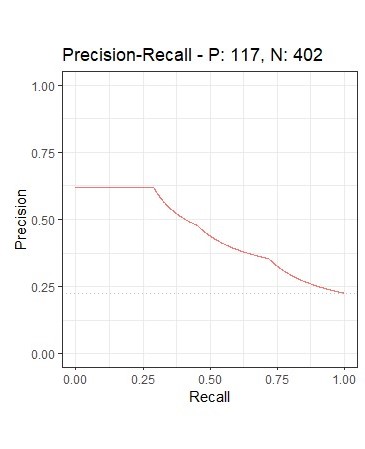


| **S1 Table. Olfactory test in relation to symptoms.** | |  |  |
| --- | --- | --- | --- |
|  | SARS-COV2 positive | SARS-COV2 negative | Odds ratio (95% CI) |
| **All patients** | N=117 | N=402 |  |
| Test 1 positive | 74 (63.2) | 193 (48) | 1.86 (1.22-2.85) |
| No smell at all | 13 (11.1) | 12 (3) | 4.06 (1.8-9.17) |
| Test 1 and 2 positive | 62 (52.9) | 156 (38.8) | 1.78 (1.17-2.69) |
|  |  |  |  |
| **Symptomatic patients** | N=104 | N=282 |  |
| Test 1 positive | 64 (61.54) | 138 (48.94) | 1.67 (1.06-2.64) |
| No smell at all | 12 (11.5) | 12 (4.3) | 2.93 (1.27-6.76) |
| Test 1 and test 2 positive | 50 (50.0) | 114 (40.4) | 1.47 (0.94-2.32) |
|  |  |  |  |
| **Asymptomatic patients** | N= 13 | N= 120 |  |
| Test 1 positive | 10 (76.9) | 55 (45.8) | 3.94 (1.03-15.03) |
| No smell at all | 1 (7.7) | 0 (0) | - |
| Test 1 and 2 positive | 10 (76.9) | 42 (35) | 6.19 (1.62-23.73) |
| Values are n (%) |  |  |  |

|  |  |  |  |  |  |  |
| --- | --- | --- | --- | --- | --- | --- |
| **S2 Table. Relevant symptoms and Olfactory Test diagnostic values** | | | |  |  |  |
| **Symptoms** | **Sensitivity (95% CI)** | **Specificity (95%CI)** | **PPV (95% CI)** | **NPV (95% CI)** | **PLR** | **NLR** |
| Fever | 0.50 (0.41-0.6) | 0.75 (0.7-0.79) | 0.37 (0.29-0.45) | 0.84 (0.8-0.87) | 2.01 | 0.66 |
| Dry cough | 0.38 (0.3-0.48) | 0.82 (0.78-0.85) | 0.38 (0.29-0.48) | 0.82 (0.78-0.86) | 2.12 | 0.75 |
| Asthenia | 0.29 (0.21-0.38) | 0.85 (0.81-0.88) | 0.36 (0.27-0.47) | 0.8 (0.76-0.84) | 1.95 | 0.83 |
| Myalgias | 0.26 (0.18-0.35) | 0.85 (0.81-0.88) | 0.33 (0.23-0.44) | 0.8 (0.76-0.83) | 1.69 | 0.88 |
| Cephalea | 0.33 (0.25-0.43) | 0.79 (0.75-0.83) | 0.32 (0.24-0.41) | 0.8 (0.76-0.84) | 1.61 | 0.84 |
| diarrhoea | 0.3 (0.22-0.39) | 0.8 (0.75-0.83) | 0.3 (0.22-0.39) | 0.8 (0.75-0.83) | 1.47 | 0.88 |
| OD | 0.16 (0.1-0.24) | 0.97 (0.95-0.98) | 0.59 (0.41-0.76) | 0.8 (0.76-0.83) | 5.01 | 0.87 |
| GD | 0.21 (0.14-0.3) | 0.96 (0.93-0.97) | 0.58 (0.42-0.73) | 0.81 (0.77-0.84) | 4.76 | 0.82 |
| **Symptoms combination** |  |  |  |  |  |  |
| OD and GD | 0.26 (0.19-0.35) | 0.94 (0.91-0.96) | 0.56 (0.42-0.7) | 0.81 (0.78-0.85) | 4.43 | 0.78 |
| Fever and dry cough | 0.64 (0.55-0.73) | 0.64 (0.59-0.68) | 0.34 (0.28-0.41) | 0.86 (0.81-0.9) | 1.77 | 0.56 |
| Fever. dry cough and OD | 0.7 (0.61-0.78) | 0.62 (0.57-0.67) | 0.35 (0.29-0.42) | 0.88 (0.83-0.91) | 1.85 | 0.48 |
| **Olfactory test results** |  |  |  |  |  |  |
| Test 1 positive | 0.63 (0.54-0.72) | 0.52 (0.47-0.57) | 0.28 (0.22-0.33) | 0.83 (0.78-0.87) | 1.32 | 0.71 |
| Test 1 and 2 positive | 0.53 (0.44-0.62) | 0.61 (0.56-0.66) | 0.28 (0.23-0.35) | 0.82 (0.77-0.86) | 1.37 | 0.77 |
| PPV Positive predictive value; NPV Negative predictive value; PLR Positive likelihood ratio; NLR Negative likelihood ratio | | | | |  |  |
